# Comparison of results of autologous versus homologous blood transfusion in open-heart surgery

**DOI:** 10.5830/CVJA-2013-020

**Published:** 2013-06

**Authors:** Bilgehan Savas Oz, Gokhan Arslan, Erkan Kaya, Celalettin Gunay, Faruk Cingoz, Mehmet Arslan

**Affiliations:** Department of Cardiovascular Surgery, Gulhane Military Academy of Medicine, Ankara, Turkey; Department of Cardiovascular Surgery, Gulhane Military Academy of Medicine, Ankara, Turkey; Department of Cardiovascular Surgery, Gulhane Military Academy of Medicine, Ankara, Turkey; Department of Cardiovascular Surgery, Gulhane Military Academy of Medicine, Ankara, Turkey; Department of Cardiovascular Surgery, Gulhane Military Academy of Medicine, Ankara, Turkey; Department of Cardiovascular Surgery, Gulhane Military Academy of Medicine, Ankara, Turkey

**Keywords:** autologous blood usage, open-heart surgery, postoperative morbidity

## Abstract

**Background:**

The aim of this study was to determine a method to decrease the use of homologous blood during openheart surgery using a simple blood-conservation protocol. We removed autologous blood from the patient before bypass and used isovolumetric substitution. We present the results of this protocol on morbidity and mortality of surgery patients from two distinct time periods.

**Methods:**

Patients from the two surgical phases were enrolled in this retrospective study in order to compare the outcomes using autologous or homologous blood in open-heart surgery. A total of 323 patients were included in the study. The autologous transfusion group (group 1) comprised 163 patients and the homologous transfusion group (group 2) 160 patients. In group 1, autologous bloods were prepared via a central venous catheter that was inserted into the right internal jugular vein in all patients, using the isovolumetric replacement technique. The primary outcome was postoperative in-hospital mortality and mortality at 30 days. Secondary outcomes included the length of stay in hospital and in intensive care unit (ICU), time for extubation, re-intubations, pulmonary infections, pneumothorax, pleural effusions, atrial fibrillation, other arrhythmias, renal disease, allergic reactions, mediastinitis and sternal dehiscence, need for inotropic support, and low cardiac-output syndrome (LCOS).

**Results:**

The mean ages of patients in groups 1 and 2 were 64.2 ± 10.3 and 61.5 ± 11.6 years, respectively. Thirty-eight of the patients in group 1 and 30 in group 2 were female. There was no in-hospital or 30-day mortality in either group. The mean extubation time, and ICU and hospital stays were significantly shorter in group 1. Furthermore, postoperative drainage amounts were less in group 1. There were significantly fewer patients with postoperative pulmonary complications, pneumonia, atrial fibrillation and renal disease. The number of patients who needed postoperative inotropic support and those with low cardiac output was also significantly less in group 1.

**Conclusion:**

Autologous blood transfusion is a safe and effective method in carefully selected patients undergoing cardiac surgery. It not only prevents transfusion-related co-morbidities and complications but also enables early extubation time and shorter ICU and hospital stay. Furthermore, it reduces the cost of surgery.

## Abstract

Cardiac surgery is one of the major fields necessitating blood transfusion. Increasing numbers of cardiac surgery cases and the requirement of large amounts of homologous blood and blood products has long been a deterrent to this form of surgery. This has forced surgeons to reduce their requirement for blood and blood products for cardiopulmonary bypass procedures and related surgical techniques.^1^,^2^ Negative outcomes using homologous blood transfusion, such as haemolytic, allergic and febrile reactions, infections (hepatitis, cytomegalovirus, HIV), renal problems, and transfusion-related acute lung injury have cause severe morbidity and even mortality in some cases.^3^,^4^

Several blood-conservation strategies have therefore evolved, with major advances being achieved from pre-donation of autologous blood, removal of autologous blood before bypass and use of isovolumetric substitution, re-infusion of the volume remaining in the extracorporal circuit, and autotransfusion of the shed mediastinal drainage blood.^5^ Other techniques, using various devices for intra-operative haemofiltration and haemoconcentration with cell separators, have been shown to decrease homologous blood usage in cardiac surgery.^6^

In this study we present our results on the use of homologous blood with a simple blood-conservation protocol. This involved removal of autologous blood before bypass and isovolumetric substitution. The morbidity and mortality outcomes of this protocol were compared in patients from two different surgical eras.

## Methods

Patients underwent isolated coronary artery bypass graft (CABG) surgery at the Department of Cardiovascular Surgery, Gulhane Military Academy of Medicine. Before 2008, CABG surgery was performed using homologous blood; thereafter we began using autologous blood.

According to the timeline, patients were retrospectively divided into two groups. A total of 323 patients were included in the study. The mean ages of the patients in groups 1 and 2 were 64.2 ± 10.3 and 61.5 ± 11.6 years, respectively. Thirty-eight of the patients in group 1 and 30 in group 2 were female. Mean NYHA class of the patients in both groups was II. The autologous transfusion group (group 1) comprised 163 patients and the homologous transfusion group (group 2) 160 patients.

Exclusion criteria were: patients with renal disease (creatinine > 1.5 mg/dl), liver disease, coagulation disorders, anaemia (haematocrit < 40.5%), low ejection fraction (EF) (30%), pre-and postoperative infection, patients who underwent emergency operations and operations other than CABG surgery. Patients with re-operations and revisions were excluded from the study.

All patients were operated on under general anaesthesia using a midline sternotomy and cardiopulmonary bypass, with membrane oxygenators and a crystalloid priming solution. A left internal mammary artery graft was used in all patients.

In group 1, autologous bloods were prepared via a central venous catheter that was inserted into the right internal jugular vein in all patients, using the isovolumetric replacement technique. The protocol for blood conservation in elective coronary surgery was as follows: cessation of antiplatelet drugs seven days before the surgery; removal of autologous blood before bypass for re-transfusion after bypass; intra-operative re-transfusion of the oxygenator and tubing contents with the help of a leukocyte filter; and adequate rewarming of patients and control of systemic blood pressure. In group 2, homologous bloods were used.

The primary outcome was postoperative in-hospital mortality and mortality at 30 days. Secondary outcomes included the length of hospital and intensive care unit (ICU) stay, time for extubation, re-intubations, pulmonary infections, pneumothorax, pleural effusions, atrial fibrillation, other arrhythmias, renal disease, allergic reactions, mediastinitis and sternal dehiscence, need for inotropic support, and low cardiac-output syndrome (LCOS).

Pulmonary infections included pneumonia and bronchitis. Pneumonia was defined by radiological evidence of new infiltration, consolidation or cavity, and antibiotic use in the presence of one of the three following criteria: purulent sputum, positive blood culture or positive bronchial secretion culture. Bronchitis was defined by the presence of purulent sputum production and antibiotic use. Pleural effusion was included in the analysis only if it required drainage during hospitalisation.

Arrhythmias other than atrial fibrillation included supraventricular arrhythmias, atrio-ventricular block requiring pacemaker, ventricular tachycardia, ventricular fibrillation and asystole. LCOS was considered when postoperative inotropic support was used for more than 24 hours. Renal failure was defined as an abnormal increase in serum creatinine levels and a decrease in urinary output.

## Statistical analysis

Statistical analysis was performed with SPSS 15.0 for Windows. Continuous data were presented as mean ± SD. Nominal data were presented as frequencies and percentages. Differences were analysed with the Levene’s test, Fischer’s exact test, Mann–Whitney *U*-test and chi-square test.

## Results

There was no difference between the two groups with regard to co-morbidities and other surgical risk factors. Patient characteristics are summarised in Table 1. Mean pre-operative haematocrit levels in groups 1 and 2 were 42.2 ± 3.9 and 41.7 ± 4.1%, respectively.

**Table 1:** Patients’ Characteristics

*Variable*	*Group 1 (n = 163)*	*Group 2 (n = 160)*	p*-value*
Age (years)	64.2 ± 10.3	61.5 ± 11.6	0.034
BMI (kg/m^2^)	25.7 ± 3.3	27.6 ± 3.0	0.045
Gender			
Male	125	130	0.033
Female	38	30	
NYHA class	2.0 ± 0.3	2.1 ± 0.3	0.062
Hypertension	65	80	0.068
Diabetes	23	45	0.002
Hyperlipidaemia	20	9	0.037
COPD	7	8	0.764
Smoking	54	50	0.718

BMI: body mass index, NYHA: New York Heart Association, COPD: chronic obstructive pulmonary disease.

There was no in-hospital or 30-day mortality in either group. There were no significant differences between the two groups related to intra-operative parameters such as cross-clamping time and cardiopulmonary bypass time. There was also no statistically significant difference in postoperative haematocrit level between the groups. However the mean extubation time, ICU and hospital stays were significantly shorter in group 1. Furthermore, postoperative drainage amounts were less in group 1 (375.8 ± 114.2 vs 543.7 ± 268.4 ml, respectively). Intra-operative and postoperative data are summarised in Table 2.

**Table 2:** Intra- And Postoperative Data

*Variable*	*Group 1 (n = 163)*	*Group 2 (n = 160)*	p*-value*
Cross-clamp time (min)	63.6 ± 21.4	64.8 ± 27.1	0.084
CPB time (min)	102.3 ± 32.0	116.3 ± 25.2	0.062
Extubation time (h)	5.6 ± 1.1	6.34 ± 1.4	< 0.01
Drainage (ml)	375.8 ± 114.2	543.7 ± 268.4	< 0.01
ICU stay (h)	23.0 ± 0.9	32.4 ± 20.2	< 0.01
Blood transfusion (intra-operative) (units)	0.3 ± 0.4	1.1 ± 0.6	0.025
Blood transfusion (postoperative) (units)	1.7 ± 0.7	2.1 ± 0.9	0.018
Hospital stay (days)	7.0 ± 1.1	8.7 ± 3.1	< 0.01
Preoperative haematocrit (%)	42.2 ± 3.9	41.7 ± 4.1	0.628
Discharge haematocrit (%)	33.8 ± 2.9	31.8 ± 2.3	0.002

CPB: cardiopulmonary bypass.

There were significant differences in postoperative morbidities. Significantly fewer patients had postoperative pulmonary complications, pneumonia, atrial fibrillation and renal disease. The number of patients who needed postoperative inotropic support and those with low cardiac output were also significantly lower in group 1. Data related to postoperative morbidities are detailed in Table 3.

**Table 3:** Postoperative Morbidity Data Of The Groups

*Variable*	*Group 1 (n = 163)*	*Group 2 (n = 160)*	p*-value*
Re-intubation	0	0	1
Sternal infections	2	6	0.148
Pulmonary complications	5	24	< 0.001
Pneumonia	1	6	0.054
Atrial fibrillation	6	26	< 0.001
Renal disease	4	8	0.231
Inotropic support	2	15	0.001
LCOS	2	9	0.003
30-day mortality	0	0	1

LCOS: low cardiac output syndrome

## Discussion

Blood donation is problematic globally, largely due to donorrelated factors, which may differ from country to country. In a study by Kubio *et al.*,^7^ among donors from Ghana, positive rates for infectious disease markers were 7.5% for hepatitis B surface antigen, 6.1% for hepatitis C virus, 3.9% for human immunodeficiency virus and 4.7% for syphilis. This amounted to 22.2% of the available donors being rejected due to infectious diseases, or one in every five donors.

In another study by Madrona *et al.*,^8^ the main reasons for donor refusal were listed among 2 070 patients as deficiency anaemias (3%), major surgery (3.6%), minor surgery (1.9%), high-risk behaviour (1.0%), drug or alcohol consumption (0.3%), more than three or four donations in a year (3.5%), pregnancy or lactation (1.2%), endoscopy, tattoo and piercings (10.5%), fever, mild infections (15.4%), hypotension (1.3%), malaise, unwell (11.0%), delivery, miscarriage (2.2%), unreliable answers (0.5%), tachycardia or bradycardia (3.6%), blood pressure > 180/> 110 mmHg (6.4%), and taking medication (7.4%). Nearly three out of every four donors in this study (72.8%) were considered unsuitable.

In cardiac surgery patients, postoperative bleeding may be considerable despite meticulous operative technique. In some regions it may be difficult to find even two units of blood for open-heart surgery. Blood-conservation strategies are therefore very important. Despite a widespread interest in reducing blood use for cardiac procedures, the practice of homologous blood transfusion is still widespread.

On average 50 to 60% of patients undergoing cardiac surgery receive blood transfusions.^9^ These patients are prone to transfusion-related morbidity and complications, such as allergies, renal disease, pulmonary complications and infection.^3^,^4^ Homologous blood usage also makes surgery more costly. There is growing evidence of an association between transfusion of blood products and increased morbidity and mortality, and reduced long-term survival rates.9

We therefore started blood-conservation protocols using autologous blood that was prepared before the bypass procedure and re-transfused into the patient at the end of surgery. In this study we compared results from two different surgical eras.

Although autologous blood transfusion has the potential to decrease bleeding following surgery, Helm *et al.*^10^ showed no statistical difference in the auto-transfusion group between the amount of autologous blood removed before the administration of heparin (1 532 ± 320 ml) compared with the amount of postoperative bleeding. However in our study, when we compared the amount of drainage in the two groups, there was a statistically significant difference in favour of group 1 (*p* < 0.01).

In their study, Paker *et al.*^11^ could not demonstrate any difference in the group with no blood or blood products used following cardiac surgery with regard to parameters of extubation time and ICU stay, compared with a group using blood/blood products. However in our study, there were significant differences between the two groups in terms of extubation time, ICU and hospital stay. These parameters were less in group 1. The number of patients needing inotropic support and patients with LCOS was also lower in group 1.

David and colleagues^12^ reported that in patients undergoing cardiac surgery, transfusion was found to be associated with an increased risk of atrial fibrillation, with an odds ratio of 1.18 for each unit of blood transfused. Sood *et al.*^13^ also found that atrial fibrillation was twice as common in transfused patients. Similarly, in our study, the number of patients with postoperative atrial fibrillation was higher in group 2.

Gökşin *et al.*^14^ showed the beneficial effect of autologous blood transfusion with regard to lung damage following ischaemia–reperfusion injury. Although not directly related, in our study when compared to group 2, there were fewer pulmonary complications in group 1 following cardiac surgery. This may have been related to the earlier extubation and less time in ICU observed in group 1 patients. Shorter ICU and hospital stay in group 1 may also have been due to the lack of early complications related to homologous blood transfusion.

Using autologous blood transfusion reduces the cost of surgery. In our hospital, one unit of fresh whole blood costs 60 Turkish Lira (TL) (nearly 25 Euro) and one unit of erythrocyte suspension costs 90 TL (nearly 38 Euro). In group 1, 328 units of blood and blood products were used, and in group 2, 517 units. Comparing costs, it was 16 480 TL (nearly 7 012 Euros) in group 1 and 41 730 TL (nearly 17 757 Euros) in group 2. The difference was more than 10 000 Euros. It is easy to see the cost effectiveness of using autologous blood transfusion.

## Conclusion

A simple and inexpensive blood-conservation programme, mainly combining autologous blood removal before bypass and re-transfusion of the volume remaining in the oxygenator, has enabled us to avoid homologous transfusions. Autologous blood transfusion is a safe and effective method in selected patients undergoing cardiac surgery. It not only prevents transfusion-related co-morbidities and complications but also allows earlier extubation time, and shorter ICU and hospital stay. Furthermore, it reduces the cost of surgery.
